# A Multi-Analytical Study of Nanolignin/Methylcellulose-Coated Groundwood and Cotton Linter Model Papers

**DOI:** 10.3390/polym17212934

**Published:** 2025-10-31

**Authors:** Mia Bloss, Marianne Odlyha, Charis Theodorakopoulos

**Affiliations:** 1 Science in Conservation of Fine Art, School of Design, Arts and Creative Industries, Northumbria University, Newcastle upon Tyne NE1 8ST, UK; 2 School of Natural Sciences, Birkbeck, University of London, London WC1E 7HX, UK

**Keywords:** lignin nanoparticles, kraft lignin, methylcellulose, paper conservation, consolidation, permanence, cellulosic materials, cultural heritage

## Abstract

This paper presents the synthesis of sustainable lignin nanoparticles (LNPs) and their application in methylcellulose (MC) as LNP/MC coatings for handmade papers. LNPs were produced from bulk kraft lignin via an acetone/water and sonication method, then incorporated into a 1 wt% methylcellulose (MC) matrix at concentrations of 0.4, 1, and 2 wt%. Groundwood and cotton linter papers were coated and exposed to 90 °C and 45% relative humidity (RH) for 16 days and the samples’ response to ageing at different concentrations of nanolignin was tested using a multi-analytical approach. The morphology of the LNPs was revealed with scanning electron microscopy, and most LNPs measured below a diameter of 30.8 nm. Colourimetry showed coated samples were inherently darker than uncoated samples but mostly stable in colour. pH remained near neutral for coated groundwood papers during ageing, but cotton papers were consistently acidic. Fourier transform infrared (FTIR) spectroscopy identified spectral similarities between uncoated and coated groundwood samples at approximately 1635 cm^−1^ and 1725 cm^−1^, attributed to carbonyl and carboxyl groups, suggesting that LNPs did not contribute to the formation of these groups during ageing. Controlled environment dynamic mechanical analysis (DMA-RH) found improved consolidation and lower elongation in most LNP/MC-treated samples. These results indicate that there may be potential for LNPs within paper conservation.

## 1. Introduction

Paper is a heterogeneous material composed primarily of cellulose, hemicellulose, and lignin. Among these, lignin occupies a contentious position in paper conservation research and practice. The prevailing belief among conservators is that lignin, whether present in high concentrations in groundwood paper or in trace amounts, is a key contributor to paper’s rapid degradation [1]. However, conservation research has challenged this assumption, showing that lignin-rich pulps do not necessarily exhibit greater mechanical deterioration after ageing when compared to lignin-free pulps [2,3,4]. Historically, other factors have been implicated in the brittleness of wood pulp papers. Notably, the nineteenth century Western paper industry’s reliance on low-quality overbeaten and impure pulps and the widespread use of acidic alum-rosin sizing in the nineteenth century—concurrently with the rise of wood pulp paper—may have resulted in hydrolysis of cellulosic and wood pulp papers, leading to chemical deterioration and mechanical failure [5,6,7]. Therefore, lignin continues to be viewed negatively, although preservation-focused research questions its direct role in paper embrittlement.

Outside conservation, lignin is increasingly valorised for its sustainability potential over the past decade. The paper industry generates over 70 million tons of kraft lignin waste annually, with 95% incinerated for fuel [8,9,10]. Lignin’s intrinsic properties, including thermal stability, high tensile strength, crystallinity, antimicrobial effects, and free-radical scavenging, make it a promising material for advanced materials, including composite coatings and films [11,12,13,14,15,16,17,18,19]. However, its irregular bulk structure and characteristics limit its utility. Instead, lignin nanoparticles (LNPs) circumvent these issues by taking advantage of its hydrogen-bonding and π-stacking interactions, offering a more reactive and stable alternative suitable for integration into polymer networks [20,21].

Nanomaterials, particularly nanocellulose, have garnered attention in recent years within paper conservation [22]. Nanocomposites are of particular interest due to their reinforcing capabilities. For example, nanocellulose incorporated into thin films prepared from 1 wt% aqueous methylcellulose dispersions improved the films’ water vapour barrier properties and thermal stability [23]. Similarly, starch/LNP composite coatings have demonstrated improved barrier and thermal properties in paper packaging applications [24].

In conservation, new materials are often evaluated in terms of their practical performance and directly compared to those already established in the field. Nanoparticle research exemplifies this comparative approach. For instance, Poggi et al. (2010) [25] demonstrated that using magnesium hydroxide nanoparticles improved paper pH more effectively than the magnesium oxide particles used in a commercial paper deacidification spray. Similarly, Giorgi et al. (2005) [26] assessed magnesium hydroxide nanoparticles against the Wei T’o mass deacidification method by measuring the degree of polymerisation (DP) in treated samples. Beyond deacidification, micro- and nanoscale polymers have exhibited promising visual properties for conservation. Microfibrillated cellulose (MFC) mending films, for example, are transparent, thin, colour-stable under UV light, and possess high tensile strength comparable to or exceeding that of traditional Asian tissue papers used in conservation mending [27]. Alongside visual assessment, Bridarolli et al. used dynamic mechanical analysis under controlled relative humidity (DMA-RH) [28,29] that enabled comparison of the mechanical behaviour of cotton canvases treated with nanocellulose versus traditional consolidants. This study revealed an enhanced consolidation and responsiveness to humidity cycling compared to untreated samples.

To evaluate a new material, it is essential to examine a broad range of properties and a multi-analytical approach is particularly valuable. Polarised light microscopy (PLM) and scanning electron microscopies (SEM) provide insights into the morphology of the nanomaterials, which can inform interpretations of mechanical behaviour [30]. DMA-RH demonstrates whether an increase in stiffness occurs and how programmed changes in relative humidity affect stiffness, consolidation, and elongation in paper fibres [29,31]. Properties that influence the longevity of paper, such as acidity and colour change, commonly referred to in conservation literature, are both indicators of underlying chemical changes [27,32,33,34,35]. Chemical characterisation methods like attenuated total reflectance Fourier transform infrared spectroscopy (ATR/FTIR) are instrumental in monitoring these changes.

To date, the use of LNPs in conservation has been largely limited to the consolidation of archaeological woods [36,37]. One of the few studies exploring LNPs for paper consolidation noted that incorporating up to 10 wt% lignin content in nanocellulose films reduced colour change in paper after humid heat and UV light ageing [38]. However, the findings are complicated by the use of a single commercial, mixed fibre, gelatine-sized cellulosic paper as a substrate and an ethyl methacrylate–methyl acrylate copolymer coating as a control that is rarely used in paper conservation [39].

This paper offers a preliminary investigation on the potential of kraft LNPs for paper conservation. By applying LNPs in aqueous methylcellulose to model handmade cotton linter and groundwood papers, the study evaluates their impact on paper permanence and mechanical strength. This study ultimately questions whether the beneficial properties of lignin observed in other disciplines align with the conservation emphasis on the preservation of paper. Focusing on indicators such as pH, colourimetry, responsiveness to fluctuating humidity, improvement in mechanical properties, and chemical changes associated with oxidation, alongside the coverage of coatings on the paper fibres, this study offers a preliminary evaluation of the interaction of LNP/MC composites with paper substrates. 

## 2. Materials and Methods

### 2.1. Materials

Alkali (kraft) lignin (Sigma-Aldrich, St. Louis, MO, USA, CAS No. 8068-05-1) was purified twice before use. Acetone (Sigma-Aldrich, St. Louis, MO, USA, CAS No. 67-64-1), Methocel™ A4C Methylcellulose, MC, (Dupont, Wilmington, DE, USA, CAS No. 9004-67-5), calcium hydroxide, Ca(OH)_2_, (Sigma-Aldrich, St. Louis, MO, USA, CAS No. 1305-62-0), potassium carbonate, K_2_CO_3_, (Sigma-Aldrich, St. Louis, MO, USA, CAS No. 584-08-7), Cargille Meltmount™ (Cargille, Cedar Grove, NJ, USA, 1.662, code 5870), 9 mm PELCO Tabs Carbon Conductive Tabs, Double Coated (agar Scientific, Rotherham, UK, AGG3357S), and silver conductive paint (Electrolube, Woking, UK, part no. SCP03B) were used as received. Cotton linters (George Weil & Sons, Guildford, UK) and unused, unprinted groundwood newspaper (Atlantis Art, London, UK) were used as fibre sources. 

### 2.2. Lignin Purification

A volume of 250 mL of deionised (DI) water was heated to 70 °C on a hot plate (Jenway, Sheung Wan, Hong Kong, model 1000) in a 500 mL Erlenmeyer flask following recommendations by Silva et al. (2024) [40]. With a magnetic stir bar, 25 g of lignin (more than the required amount for preparation of LNP solutions; ultimately only 8.0 g of lignin was used. See Section 2.3 below) was stirred into this preheated water for 15 min at 400 rpm. Filter paper (Fisherbrand, Waltham, MA, USA, QL100, 90 mm) was dampened in a Buchner funnel. An aliquot of lignin in water was placed into the centre of the filter paper and vacuum was applied with a water aspirator [41]. All washed lignin was dried in a vacuum oven (Asynt 31 L Vacuum Oven, Isleham, UK) for 24 h at 60 °C [42]. The dried lignin was stored in a desiccator away from light until used.

### 2.3. Preparation of Nanolignin Solutions 

Kraft lignin nanoparticles (LNPs) were synthesised primarily with an acetone/water cosolvent approach [42]. Acetone (140 mL) and DI water (60 mL) (25 °C) was added to washed, dry kraft lignin (8.0 g) in a 500 mL Erlenmeyer flask. This was stirred with a magnetic stir bar for 3 h at 400 rpm (Jenway, Sheung Wan, Hong Kong, model 1000). After stirring, 10 mL of this solution was pipetted over the course of 1 min into a 500 mL beaker containing 200 mL of DI water (25 °C), stirring with a magnetic stir bar at 500 rpm for a further 15 min. This was repeated, producing 20 × 200 mL lignin/acetone/water dispersions, each containing approximately 0.4 g lignin [42]. The acetone was allowed to evaporate from the dispersions in ambient conditions in a fume hood for 24 h. Three of the 200 mL dispersions were set aside as excess as only seventeen were needed to produce the desired LNP concentrations. 

A probe sonicator with 0.5-inch horn (Q500 Sonicator, QSonica, Newtown, CT, USA) sonicated the dispersions in each beaker for 10 min (with pulse ‘on’ for 50 s and ‘off’ for 10 s each min) at 20 kHz and amplitude 40 while the beakers were set into ice to prevent overheating [43]. 

After sonication, the aqueous dispersions were concentrated. Placing the beakers onto large hot plates (IsotempSP88850200, Fisherbrand, Waltham, MA, USA) set to 100 °C, excess DI water was slowly removed via evaporation until ten beakers’ dispersions measured 20 mL each, five beakers’ dispersions measured 40 mL each, and two beakers’ dispersions measured 100 mL each. Each set of two, five, or ten beakers was combined by stirring for a further 15 min, so that the LNP concentration in each 200 mL volume was 0.4 wt%, 1 wt% and 2 wt%, respectively, in three 250 mL jars sealed with lids and Parafilm M (Amcor, Zurich, Switzerland).

MC powder (2 g) was manually stirred into each 200 mL dispersion of 0.4 wt%, 1 wt%, and 2 wt% LNPs after weighing with an electronic balance (TechMasterES-300H, Suzhou, China), and a fourth jar was produced with only MC at this same 1 wt% aqueous concentration. All dispersions were chilled for 36 h in a refrigerator until homogenised. Because the pH of the 0.4 wt%, 1 wt%, and 2 wt% LNP solutions measured pH 6.46, 6.25, and 6.12, respectively, a saturated solution of Ca(OH)_2_ was added dropwise with a pipette with stirring until pH reached neutral. These LNP/MC dispersions were now ready to apply to samples.

### 2.4. Sample Preparation

Model papers were produced onsite by hand without sizing or fillers. The fibres were soaked and macerated in a commercial blender (Waring NuBlend^®^ BB185S, Stamford, CT, USA), then dispersed into DI water in large tubs with an electric hand whisk (Dynamic, Mortagnes/Sèvre, France) to prevent flocculation. A wove mould was dipped into the tubs and quickly drawn out while shaking the mould evenly in both directions to avoid a prominent grain direction. Sheets were couched and air-dried overnight. The following day, the sheets were cold pressed between felts in an etching press for a smoother surface texture and denser structure.

Four samples (8 cm by 14 cm) were cut from each larger sheet in the same orientation, avoiding the deckle edge. Samples with mass outside of the acceptable range—1.0 ± 0.1 g for cotton papers and 0.8 ± 0.1 g for groundwood papers, measured with an electronic balance (TechMasterES-300H, Suzhou, China)—or having visible faults with the pulp distribution were discarded. A volume of 6 mL of each LNP/MC dispersion and MC coating—0.4 wt% LNP/1 wt% MC, 1 wt% LNP/1 wt% MC, 2 wt% LNP/1 wt% MC, and 0 wt% LNP/1 wt% MC—was applied with a synthetic brush to each side of the cotton sample papers (recto-verso), allowing the sample to dry between each coating. Due to differences in the paper fibres’ absorbance, 3 mL was applied to each side of the groundwood paper. The final 0.5 cm on each narrow edge of the sample was left uncoated to differentiate between untreated and treated substrate. 

### 2.5. Accelerated Ageing

A dry Cole-Parmer Forced Air Oven (52411-series, Cole-Parmer, St Neots, UK) capable of ambient temperatures up to 250 °C was used to age treated and untreated samples. TAPPI Test Method T 544 cm-19 (aging of paper and board with moist heat) was followed for standards on ageing duration, temperature, and relative humidity (RH) [44]. Samples were tied to linen threads and to linen tape stretched across the mouth of 5 L glass jars (Fido, Bormioli Rocco, Fidenza, Italy) to hang suspended without touching the bottom or walls of the jar as much as possible. Seventy samples out of the eighty prepared were divided between six jars, divided by fibre type and with non-LNP-containing coatings and uncoated samples sharing the same jar. 0.4 wt% LNP/MC and 1 wt% LNP/MC-coated samples were also placed in the same jar. To adjust RH, a saturated solution of K_2_CO_3_ was prepared (60 g K_2_CO_3_ in 50 g water), and one open jar of this saturated solution was placed into each larger jar. Before ageing, these prepared samples were conditioned to approximately 43% RH for 24 h [45]. Ageing proceeded at 90 °C for sixteen days total. One sample of each combination of treatment and fibre type was removed from the jars at intervals of 24 h, 48 h, 72 h, 144 h, 312 h, and 384 h.

### 2.6. pH Measurements

A cold extract method following TAPPI/ANSI T 509 om-22 (hydrogen ion concentration (pH) of paper extracts (cold extraction method) was implemented, scaled-down to conserve samples [46]. Pieces with a mass of 0.1 g measured with an electronic balance were cut from each sample. Each 0.1 g piece was placed with 7 mL of DI water (25 °C) in a watch glass-covered 250 mL glass jar, pulverised with a glass rod, and left to extract for 1 h. The pH was recorded with a LAQUAtwin pH-22 meter (HORIBA, Kyoto, Japan, accuracy pH ±0.01) and repeated in triplicate for all samples, with pH recorded three times from each extract; therefore, 0.3 g of each sample was consumed for this test, producing nine readings. If readings deviated by pH 0.1 between tubes or samples, the extraction and/or readings were repeated. All measurements were taken at 25 °C.

### 2.7. Colourimetry

A CM-2600d (Konica Minolta, Basildon, UK) spectrophotometer was used. The spectrophotometer operates with the specular component included (SCI) or excluded (SCE) and conforms to CIE No. 15, ISO 7724/1, ASTM E 1164, DIN 5033 Teil 7, and JIS Z 8722 condition and standards [47,48,49,50,51]. Fifteen SCE readings (at five locations and taken in triplicate) of the samples were averaged to accommodate for heterogeneous colour and the large dimensions of the samples. The instrument also obtained ultraviolet/visible diffuse reflectance spectra within a wavelength range from 360 to 740 nm and the CIEL*a*b* colour space factors using the SpectraMagicNX_Ver340 software. Measurements were collected in SCE mode. The CIEDE2000 formula was selected to calculate colour difference, as determined by Sharma et al. [52]. Overall, colour difference was calculated by comparing collected L*a*b* values from samples aged to various degrees to the unaged sample for each combination of fibre type and treatment.

### 2.8. Attenuated Total Reflectance/Fourier Transform Infrared Spectroscopy (ATR/FTIR) 

A Perkin Elmer Frontier SPECTRUM3 FTIR spectrometer (High Wycombe, UK, 8000−350 cm^−1^ with a best resolution of 0.4 cm^−1^) was used equipped with a UATR Diamond ATR crystal (2.4 refractive index (*n*) and 2 μm depth of penetration (*dp*) at 1000 cm^−1^). ATR/FTIR spectra of a 4000–380 cm^−1^ range were obtained after 32 scans at a 4 cm^−1^ resolution and processed with PerkinElmer Spectrum™.

### 2.9. Polarised Light Microscopy (PLM)

A Motic BA310POL polarised light microscope equipped with a Moticam A1 digital microscope eyepiece camera (Motic, Universal City, TX, USA) and MotiConnect v.1.0.1.9 software was employed to characterise the fibres and their coatings in a bright field (exposure time 100 msec to 1 s). Objective lenses were used at 4×, 10×, 40×, and 60× with a 10× ocular lens for overall magnification of 40×, 100×, 400×, and 600×.

### 2.10. Scanning Electron Microscopy (SEM)

A TESCAN MIRA 3 scanning electron microscope (SEM) (Huntingdon, UK) equipped with an Everhart-Thornley type secondary electron (SE) detector and an in-beam secondary electron (In-Beam SE) detector was used in a low vacuum mode (10 Pa). Papers cut into a few mm-sized samples were fixed on an adhesive carbon tape coated with the silver conductive paint dot, placed on metal stubs and sputter coated with a 5 nm Pt layer in a Q150V ES plus sputter coater (Quorum, Laughton, UK). The SEM system was operated with the MiraTC software at 5 kV, a working distance (WD) of 15 mm with the SE detector for view fields of 180 and 500 μm to detect the fibre morphology, and a 7–8.5 mm WD range with the In-Beam SE detector for 1.5–10 μm view fields. ImageJ v.1.54g software was used to measure the size of the LNPs from these SEM images.

### 2.11. Controlled Relative Humidity—Dynamic Mechanical Analysis (DMA-RH)

DMA-RH was used to measure the mechanical properties and how these values vary with programmed RH. Samples were clamped in tension with a free length of 5 mm in a Tritec 2000 B DMA connected to a Humidity Controller (Lacerta Technology, Shepshed, UK). Sample width was between 5–7 mm and thickness 0.2 to 0.3 mm. A sinusoidal strain was applied (0.1%) to the samples and values for elastic (or storage) modulus and displacement were obtained. All measurements were conducted at 25 °C where the temperature was controlled using a recirculating water bath. A static force (0.5 to 1.5 N) was applied to the sample to raise the applied static force above the dynamic force to prevent sampling buckling during testing. Measurements were made at frequencies (1 Hz, 10 Hz). All results are reported at a frequency of 1 Hz. The RH programme was applied starting at 20% RH for 30 min and then increasing at 4% RH/min to 80% RH where it remained for 60 min. It was then reduced at 4% RH/min to 20% RH where it remained for a further 30 min. This programme was applied to unaged and aged samples. Samples were conditioned for at least 24 h prior to testing in closed 50 mL polypropylene Corning Falcon^®^ tubes (Corning, NY, USA) with dry silica gel desiccant. 

The storage or elastic modulus (*E′*) as obtained from DMA-RH represents the amount of elastic energy that a polymer put under sinusoidal strain can store elastically. Higher *E′* indicates that a polymer can store more energy and therefore a polymer with higher *E′* represents a more elastic, stiffer material. This is a crucial metric for understanding the practical durability of treated papers, especially in environments with fluctuating RH.

Displacement, another property measured by DMA-RH, gives the dimensional change in the sample subjected to the applied sinusoidal strain. It was calculated by dividing the measured displacement by the free length of the sample (5 mm), the distance between the clamps, to determine the percent by dimensional change. While percent displacement will be presented in the results, it will be referred to as ‘displacement’ for brevity. A positive displacement relative to the samples’ length indicated elongation. If the sample did not return to its original displacement before being subjected to RH changes, then the dimensions were permanently affected by cycling RH [28]. 

## 3. Results and Discussion

### 3.1. Polarised Light Microscopy (PLM)

**Figure 1:**
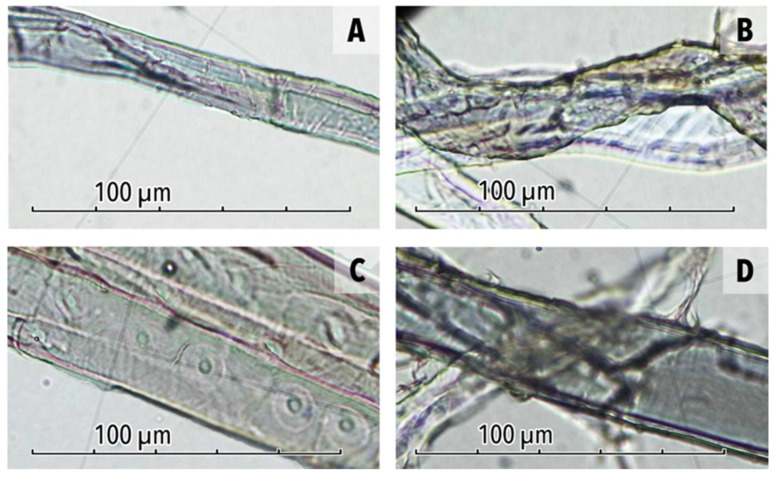
PLM photomicrographs in parallel planes of unaged, uncoated cotton fibres removed from samples. (**A**) Detail of uncoated cotton fibre, with a smooth fibre wall (**B**) Detail of 2 wt% LNP/MC-coated cotton fibre, with a rough and dark coloured fibre wall. (**C**) Detail of a light-coloured uncoated groundwood fibre, with a smooth fibre wall. (**D**) Detail of 2 wt% LNP/MC-coated groundwood fibre, dark in colouration with a rough wall.

Brightfield polarised light microscopy (PLM) of uncoated cotton and groundwood fibres in parallel planes revealed nearly colourless fibres before treatment (Figure 1A,C).

The 2 wt% LNP/MC-coated cotton fibre is darker in colouration and LNPs appear to have been deposited onto the outer surfaces of the fibres, resulting in an uneven, textured surface on the fibre walls (Figure 1B). The groundwood fibre also appears rougher and darker, albeit to a lesser extent, likely due to the smaller volume of LNP dispersion applied to the thinner, less absorbent groundwood paper (Figure 1D).

### 3.2. Scanning Electron Microscopy

**Figure 2:**
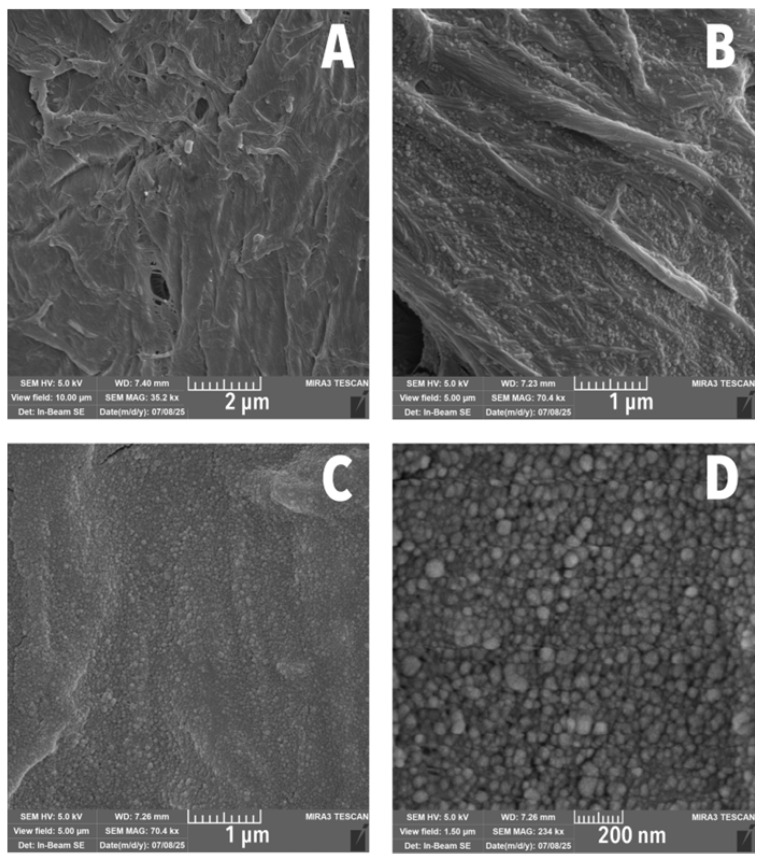
SEM images of cotton samples. (**A**) Uncoated cotton, the base fibre. (**B**) 0.4 wt% LNP/MC cotton, with LNPs filling the lowest portions of the fibres but leaving the ridges exposed. (**C**) 2 wt% LNP/MC cotton, with LNPs entirely coating the fibre, leaving a smoother topography than the 0.4 wt% LNP/MC-coated sample. (**D**) Detail of nanoparticles as viewed on 2 wt% LNP/MC cotton.

**Figure 3:**
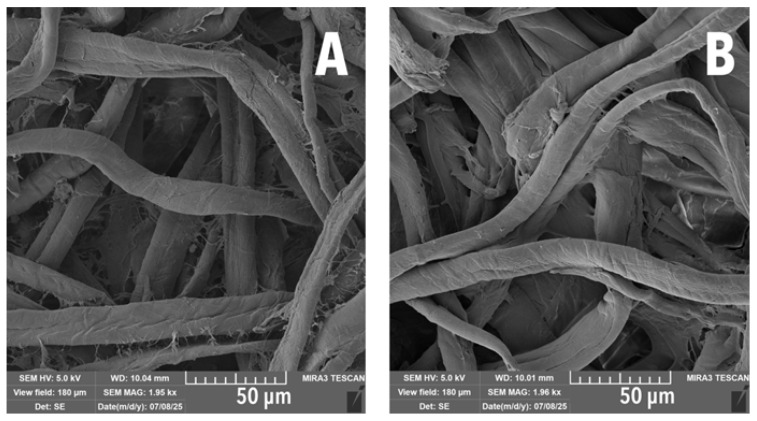
SEM images of cotton fibre clusters. (**A**) Uncoated cotton. (**B**) 2 wt% LNP/MC cotton. Fibrillated walls of fibres are much smoother, appearing as if the fibrillation had been consolidated onto the body of the fibre.

SEM showed that the surface distribution of LNPs was relatively uniform, especially at higher concentrations (Figure 2A,D). At 0.4 wt% LNP/MC, some fibres remained uncovered. However, at 2 wt% LNP/MC, the surface was fully coated, resulting in a smoother fibre appearance. Fibrillation along fibre walls appeared reduced, seemingly pressed into the fibre by the LNP/MC treatment. Although a few sparse strands of fibrillated cotton were still present, most had been consolidated (Figure 3). 

Nanoparticle size measurements taken directly on the SEM images indicated an average LNP diameter of 31.2 nm. Over half of the LNPs had diameters below 30.8 nm, while only 3.82% of non-aggregated LNPs exceeded 66.8 nm (Table 1). The LNPs were predominantly spherical, with minimal aggregation observed. Although the average size was smaller than expected based on the method described elsewhere [42], where diameters of approximately 75.9 nm were reported for LNPs prepared at the same initial concentration, the discrepancy may be attributed to the use of a sonicator instead of a homogeniser [42,53].

**Figure 4:**
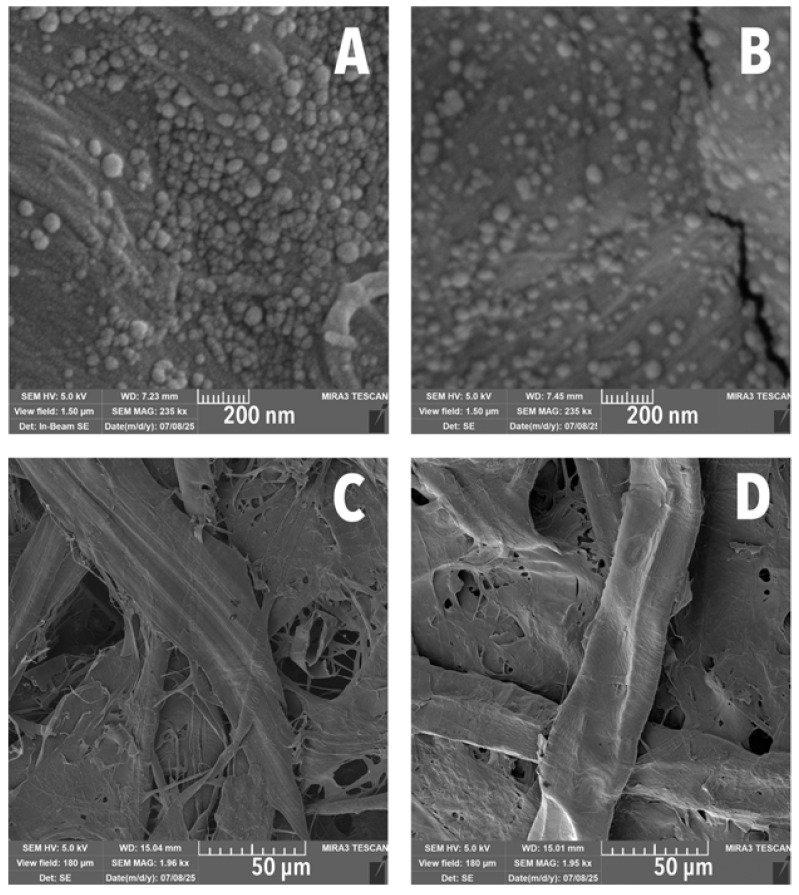
SEM images and comparison of samples with cracking to samples without. (**A**) 0.4 wt% LNP/MC cotton. (**B**) 0.4 wt% LNP/MC groundwood. (**C**) Uncoated groundwood. (**D**) 0.4 wt% LNP/MC groundwood. Cracking is visible only in LNP/MC-treated groundwoods (**B**,**D**).

The effect of the LNP/MC treatment on groundwood was most evident in the spaces between fibres. A weblike network formed, connecting the fibres with reduced fibrillation, similar to what was observed in the cotton samples (Figure 4C,D). However, unlike with treated cotton, micro-scale cracking was visible (Figure 4A,B,D), a phenomenon previously reported in lignin films by other authors [54].

### 3.3. Colourimetry

An averaged L*a*b* value was calculated for each sample, representing lightness (L*), red/green (a*), and yellow/blue (b*) metrics. The b* value is a particularly useful indicator, since increased yellowness in a material or object are undesirable [55].

**Figure 5:**
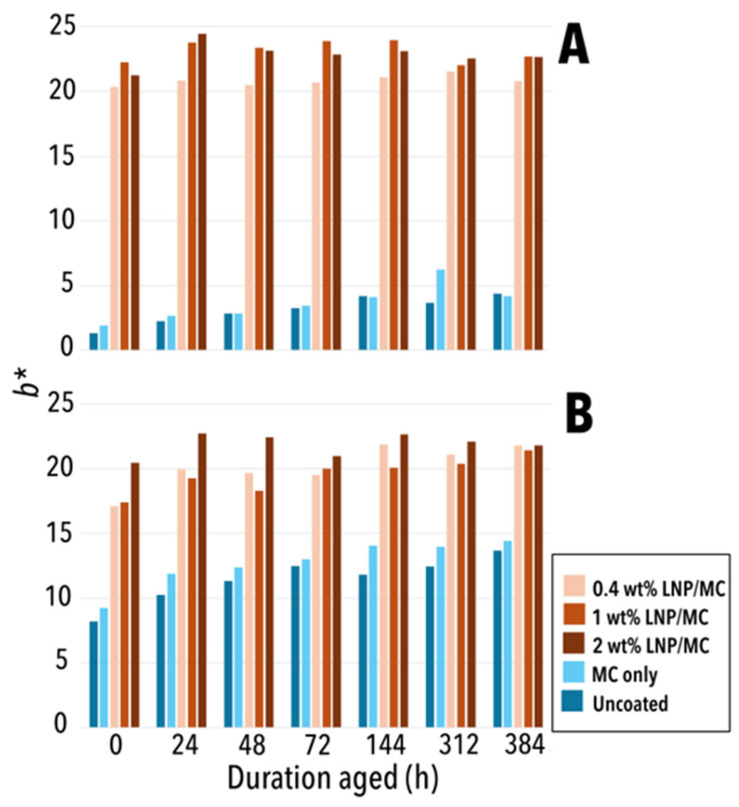
b* values for all samples, unaged to fully aged. (**A**) Cotton samples. (**B**) Groundwood samples. While uncoated and MC-coated samples gradually increase in yellowness over ageing duration, b* values never approach b* values of LNP/MC-treated samples (especially for cotton). Nevertheless, LNP/MC-treated samples are very stable in their yellowness.

In the cotton and groundwood samples, slightly higher b* values were recorded in uncoated samples and those coated only with MC, particularly after extended ageing (Figure 5). All LNP/MC-coated cotton paper samples exhibited a consistent b* value around 4, indicating a stable degree of yellowness (Figure 5A). In contrast, uncoated groundwood samples showed an increase in yellowness varies from b* values of 8.21 (unaged) to 13.65 (aged, 384 h), which is a Δb* of 5.44 (Figure 5B). Similarly, the b* values of MC-only coated samples ranged from 9.24 (unaged) to 14.4 (aged, 384 h), a Δb* of 5.17. Groundwood samples coated with LNPs/MC showed smaller increases in b* upon ageing. In particular, the 0.4 wt% LNP/MC coating resulted in Δb* = 4.74, the 1 wt% LNP/MC coating in Δb* = 4.02 and the 2 wt% LNP/MC coating in Δb* = 2.18 (Figure 5B). To sum up, samples with higher concentrations of LNPs had the smallest increases in b* after 384 h of ageing. However, even the most aged (384 h) non-LNP coated samples did not reach the lower b* values recorded on the LNP-containing samples. While this represents the limitation in using LNPs for conservation, it also highlights the potential for future experimental approaches aimed at reducing LNP colouration [56].

**Figure 6:**
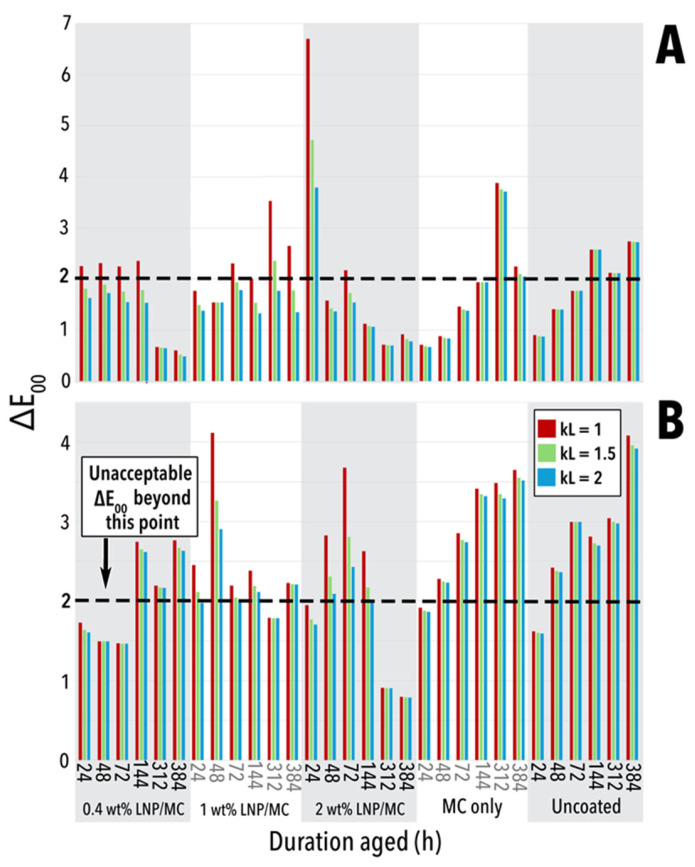
ΔE_00_ values for all samples, where colour deviation from an unaged sample is measured. Values where parametric weighting factor for lightness k_L_ = 1, 1.5, and 2 are displayed, with often significant variation in reported values depending on k_L_. Plotted ΔE_00_ values above dashed line at ΔE_00_ = 2 are considered unacceptable. (**A**) Cotton samples. (**B**) Groundwood samples.

Acceptable ΔE_00_ values for conservation purposes are often below 2.0 [57]. Values exceeding this threshold, indicating perceptible colour change, are indicated in Table S1 and marked with a dashed line in Figure 6. These instances reflect noticeable shifts in colour compared to the unaged sample for each treatment and paper substrate. Varied ΔE_00_ values at different k_L_ values of 1, 1.5, and 2 suggest differences in the paper surface texture. The k_L_ is a parametric value that accounts for surface inhomogeneity, which in this case may be associated with the coating distribution on the fibres (Figure 4A,B). When k_L_ = 1, ΔE_00_ is calculated with a perfectly smooth, featureless surface assumed. k_L_ = 2 is representative of how colours are perceived by human viewers in woods and textiles [58,59]. The greatest differences between ΔE_00_ values when k_L_ is changed from 1 to 2 were in LNP-containing treatments, suggesting that the LNPs resulted in a rougher surface, even if that roughness was occurring on the nanoscale. Interestingly, ΔE_00_ values at k_L_ = 1.5 values were closer to those at k_L_ = 1 than to k_L_ = 2, suggesting that the samples were not as inhomogeneous as typical textiles.

A noticeable colour change occurred in uncoated and MC-only groundwood samples, with significant ΔE_00_ values observed after just 24 h of ageing. In contrast, groundwood samples coated with 0.4 wt% LNP/MC had a delayed colour change, with perceptible differences emerging after 144 h of ageing. At higher LNP concentrations colour change in groundwood was less consistent but generally remained within the noticeable change range. Cotton papers demonstrated greater cotton stability overall, with lower ΔE_00_ values throughout the ageing process. However, both uncoated and MC-only coated cotton samples eventually exceeded the ΔE_00_ threshold of 2.0. Notably, some LNP-treated samples, specifically 0.4 wt% and 2 wt% LNP/MC in cotton and 1 wt% and 2 wt% LNP/MC in groundwood, initially experienced high colour change, but as ageing progressed, the colour difference diminished, and the final colour closely resembled that of the unaged LNP/MC-treated samples (Figure 6A,B).

### 3.4. Acidity—pH

**Figure 7:**
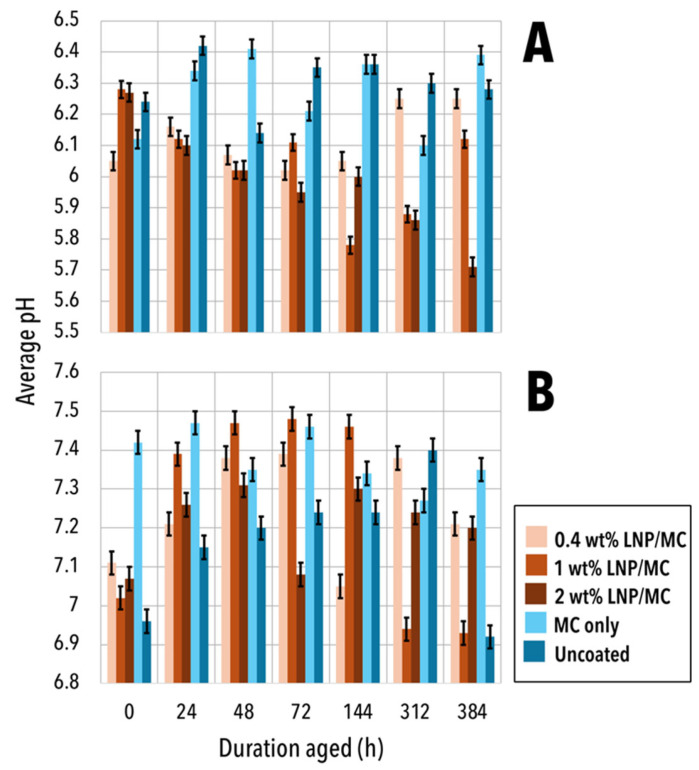
Cold extract pH of samples aged for varying durations, 0 h–384 h. (**A**) Cotton samples. (**B**) Groundwood samples. Cotton samples were always acidic, but groundwood samples sometimes entered slightly alkaline conditions. From left to right in each ageing duration (h) category, samples are 0.4 wt% LNP/MC, 1 wt% LNP/MC, 2 wt% LNP/MC, samples coated only with MC, and uncoated samples. Standard deviations are indicated by error bars.

A notable observation is that the unaged cotton paper was already slightly acidic with pH values ranging from 6 to 6.2. This is atypical for a pure cellulosic substrate and suggests that the cotton fibres had undergone some degree of oxidation prior to the LNP/MC treatment. As a result, cotton samples remained acidic throughout the tests, with average pH values ranging from 5.71 to 6.42 (Figure 7). In contrast, groundwood papers were generally neutral to slightly basic, with pH values between 6.92 and 7.48.

Across both substrates, pH trends varied slightly depending on the LNP concentration. In particular, 1 wt% LNP/MC-coated samples showed a slight decrease in pH after ageing, whereas 0.4 wt% LNP/MC-coated samples showed a slight increase in pH. For example, the pH of 0.4 wt% LNP/MC-coated cotton paper rose to pH 6.25 from pH 6.05, closely aligning with the final pH 6.28 observed in the uncoated cotton paper. Similarly, uncoated groundwood paper had a lower pH than both the 0.4 wt% and 2 wt% LNP/MC-coated groundwood samples. For both substrates, MC only-coated samples remained relatively stable in pH.

### 3.5. Fourier Transform InfraRed Spectroscopy 

#### 3.5.1. ATR/FTIR Overview

**Figure 8:**
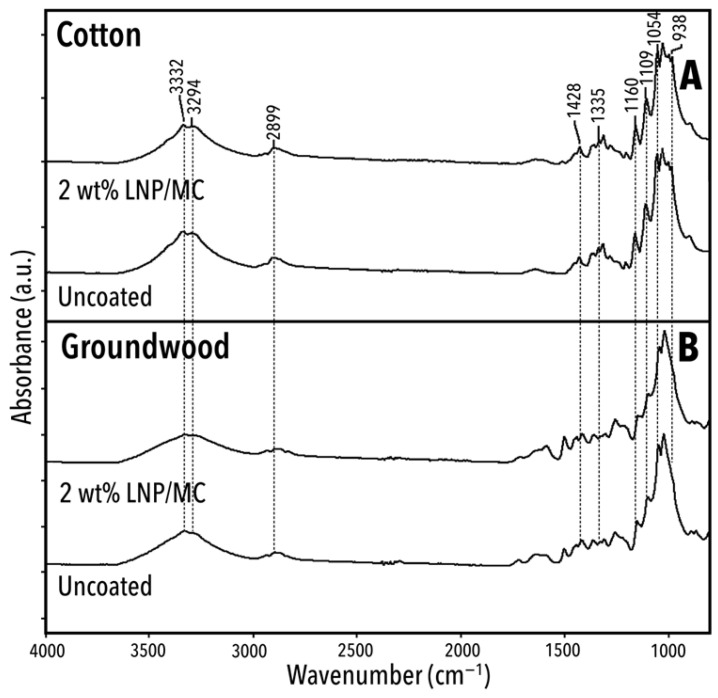
ATR/FTIR spectra for unaged samples, both uncoated and 2 wt% LNP/MC-treated. See Table 2 for discussion of these spectra. (**A**) Unaged cotton samples, 2 wt% LNP/MC-treated and uncoated. (**B**) Unaged groundwood samples, 2 wt% LNP/MC-treated and uncoated.

Figure 8 presents the spectra of uncoated and 2 wt% LNP/MC-coated cotton and groundwood model papers. A detailed overview of the significant peaks, shoulders, and bands is shown in Table 2. Most of the peaks listed in Table 2 were observed in all samples, although slight peak shifting was noted.

#### 3.5.2. Substrate-Specific FTIR Signatures and the Impact of LNP/MC Coatings

**Figure 9:**
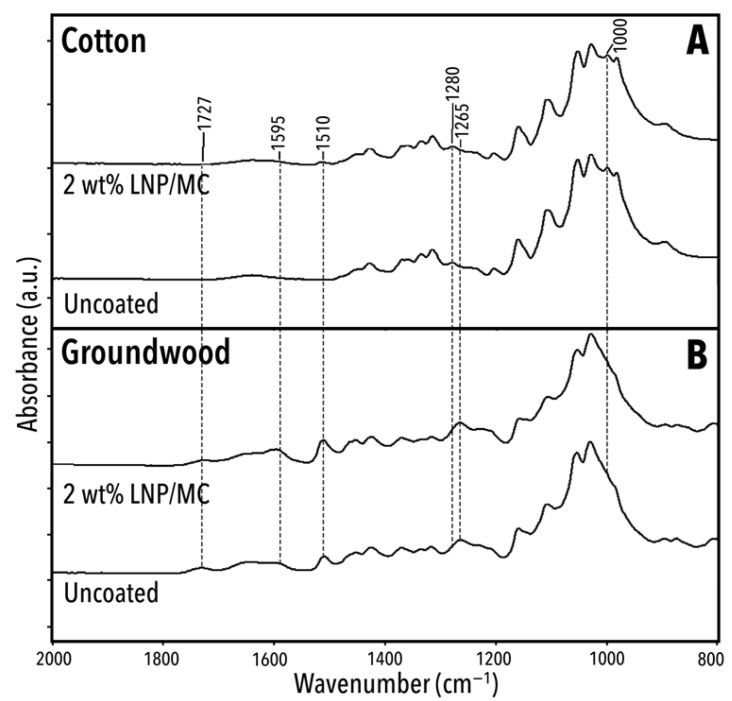
ATR/FTIR spectra for unaged samples, 2000–800 cm^−1^. Indicated with dashed vertical lines are six approximate wavenumbers at which some samples, but not all, have peaks: 1727 cm^−1^, 1595 cm^−1^, 1510 cm^−1^, 1280 cm^−1^, 1265 cm^−1^, and 1000 cm^−1^. (**A**) Unaged cotton samples, 2 wt% LNP/MC-treated and uncoated. (**B**) Unaged groundwood samples, 2 wt% LNP/MC-treated and uncoated.

Figure 9 and Table 3 present the fingerprint region of the same four unaged samples as discussed above: cotton and groundwood, uncoated and coated with 2 wt% LNP/MC, and highlights six wavenumbers where one or more significant peaks are absent from at least one sample. These missing peaks are likely due to either the LNP/MC treatment or differences in substrate composition, and they help illustrate the chemical changes introduced by the coatings and the chemical differences in the substrate fibres.

The cotton samples had bands at 1000 cm^−1^ and 1280 cm^−1^, both attributed to CH- bending vibrations in cellulose [71,72]. These bands were not present in the groundwood papers. Since the 1280 cm^−1^ band is only present in celluloses with high crystallinity [72], the spectra herein indicate that the cellulose found in the cotton paper was likely highly crystalline.

Both untreated and treated groundwood samples had detectable bands at approximately 1722–1727 cm^−1^ and 1265 cm^−1^ (Figure 9). The peak at 1722–1727 cm^−1^ is indicative of C = O stretching in lignin or hemicellulose [66,67] and the peak at 1265 cm^−1^ of phenol groups or guaiacyl (G) rings found in lignin [61,62]. Since the 1265 cm^−1^ band appeared in the uncoated groundwood sample but not in the LNP/MC-treated cotton sample, it is likely associated with the lignin content of the groundwood paper rather than the lignin introduced via the LNP/MC coating.

**Figure 10:**
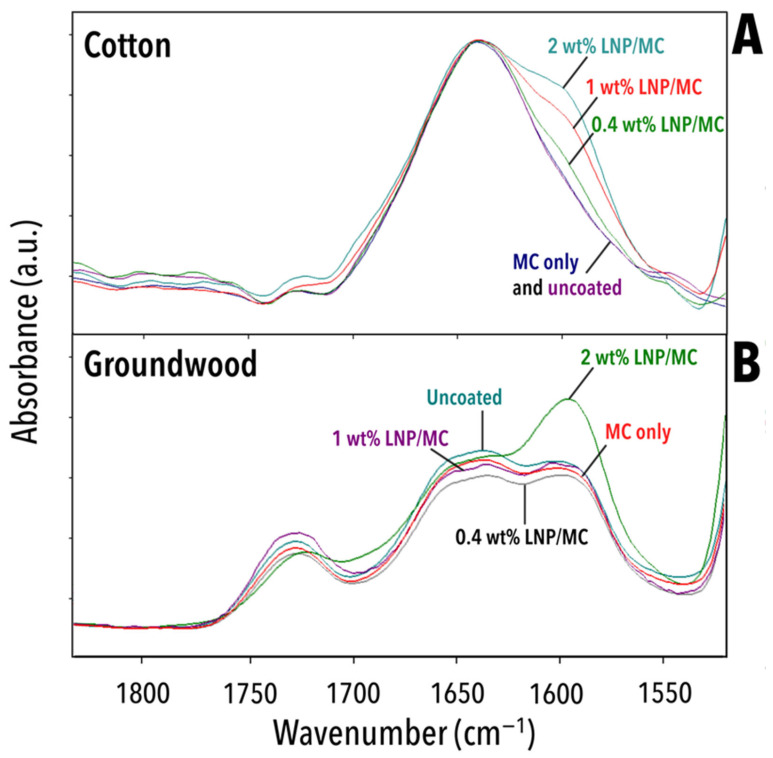
ATR/FTIR spectra for all unaged samples. (**A**) Cotton, 1850–1530 cm^−1^. (**B**) Groundwood, 1840–1435 cm^−1^.

Two new bands appeared in cotton samples after treatment: 1592–1606 and 1509–1514 cm^−1^ (Table 3). Both bands are attributable to the chemical structure of lignin. A spectral detail near 1595 cm^−1^ due to C=C and C-H stretching in the aromatic syringyl or S-type rings [64,69] in the LNPs is provided in Figure 10. In the unaged cotton samples, the 1595 cm^−1^ band increased in absorbance with increasing LNP concentration (Figure 10A). In the unaged groundwood samples, this same relationship did not exist and the samples’ absorbance at this band was not ordered by increasing LNP content. This indicates that the LNP/MC coating was detected on the groundwood papers [60,62,73], but implied that the added lignin was unevenly distributed in the sheet. If the distribution of lignin had been homogeneous, adding LNPs in any amount would have increased the peak proportionally, as was observed in cotton papers.

#### 3.5.3. The Effect of the LNP/MC Coatings on Oxidation of the Substrates

**Figure 11:**
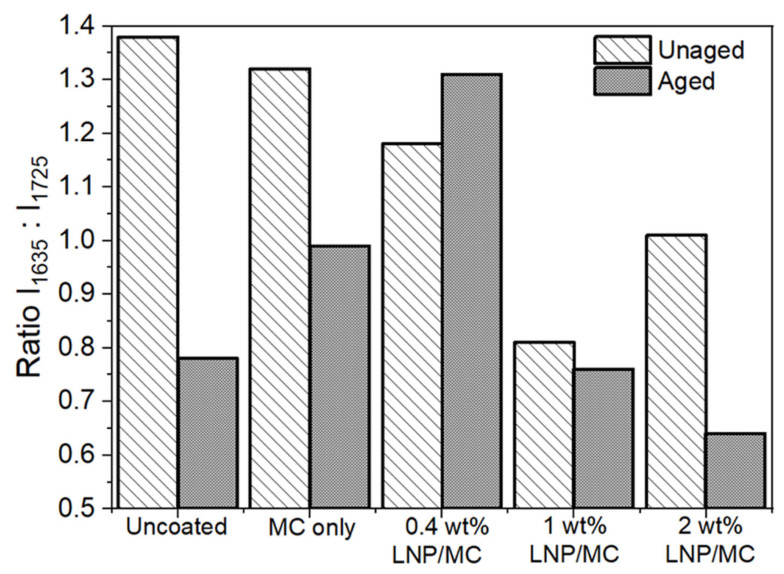
Oxidation index, or ratio of integrated areas from 1700 to 1615 cm^−1^ (I_1635_) and from 1785 to 1700 cm^−1^ (I_1725_), for unaged and fully-aged groundwood samples. All aged samples apart from one had a ratio favouring carboxyl group content which suggested that formation of carboxyl groups was not dependent on LNP content and rather was due to deterioration of base groundwood fibres.

The ATR/FTIR study also enabled monitoring of the oxidation and deterioration of the groundwood paper through the ratio of carbonyl (1725 cm^−1^) to aldehyde or carboxyl (1635 cm^−1^) bands. Following the oxidation index approach proposed by Łojewska et al. (2005) [74], the spectra were scaled to the CH stretching vibration band at 2900 cm^−1^ and integrated areas were taken of the peaks at approximately 1635 cm^−1^ and 1725 cm^−1^ to reflect the proportion of carbonyl groups to aldehyde or carboxyl groups, respectively (Figure 11). Due to the absence of the 1725 cm^−1^ band in the cotton papers, even after ageing, it was not possible to calculate the oxidation index for those samples. This observation is particularly noteworthy, as it indicates that ageing did not lead to an increased presence of carboxyl groups in the LNP-containing cotton samples.

In the groundwood papers, the oxidation ratio declined from above one for the unaged samples to below one for nearly all aged samples, indicating that ageing promoted the formation of carboxyl groups more than carbonyl groups, regardless of treatment. However, since this formation occurred equally in the uncoated groundwood paper samples, it suggests that the carboxyl groups originated from the base fibres rather than from the LNP/MC coating. Importantly, the oxidation ratio for 0.4 wt% LNP/MC-treated groundwood, unlike the rest of the groundwood samples, increased after ageing and remained over one (Figure 11). This suggests that 0.4 wt% LNP/MC may have had a protective quality against the formation of carboxylic groups during the thermal ageing process, better than MC or uncoated groundwood was able to achieve. The lack of a carboxyl peak in cotton papers, even those treated with LNP/MC coatings and aged, and the ability of the 0.4 wt% LNP/MC-treated groundwood to resist carboxyl group formation are two properties that suggest LNPs may be beneficial to the preservation of some papers.

### 3.6. DMA-RH

**Figure 12:**
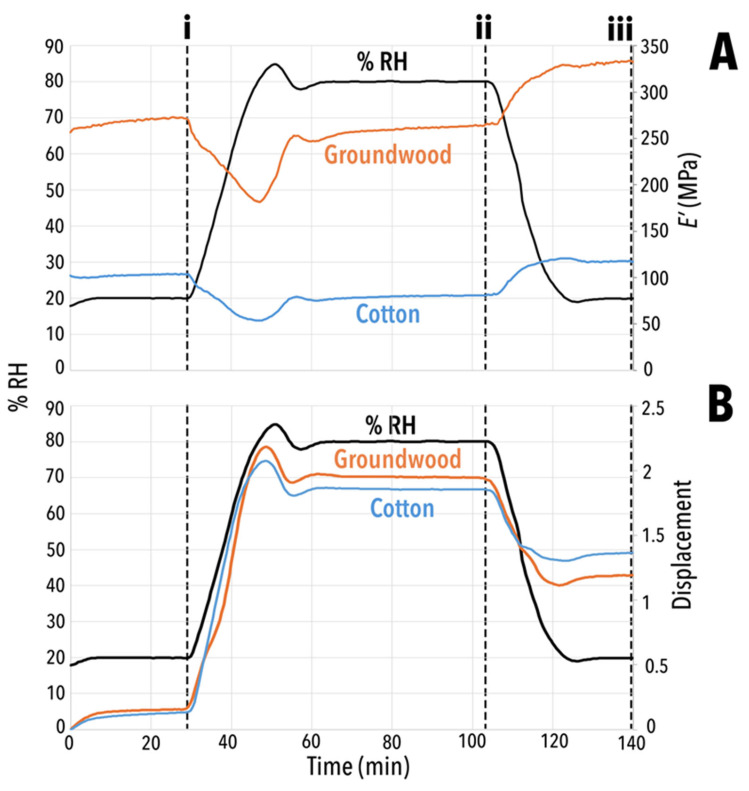
Plots of (**A**) storage modulus (*E′*) unaged, uncoated control cotton (blue) and groundwood (orange) and RH (black) vs. time (min) and (**B**) displacement vs. time (min) for papers. Some overshoot in humidity controller near 50 min is visible in the sudden spike in RH. Dashed vertical lines (i,ii) indicate ends of plateaux and the approximate times where measurements were taken for *E′* and displacement. (i) End of initial 20% RH plateau. (ii) End of 80% RH plateau. (iii) End of final 20% RH plateau.

Generally, with reference to Figure 12A, *E′* was steady in initial dry conditions (20% RH) for unaged, uncoated cotton and groundwood papers. As RH increased, *E′* decreased in value. Approaching 80% RH, a brief overshoot by the RH controller to ~85% RH caused a temporary dip in *E′*, but equilibrium was quickly restored at 80% RH due to the plasticisation from moisture. At the maximum 80% RH, the modulus, *E′_80_*, reached equilibrium and plateaued before increasing as water was removed in the drying process during the return to 20% RH to *E′_20f_*. Higher *E′_20f_* values compared to initial *E′_20i_* revealed that the uncoated control samples underwent permanent change (Figure 12A) [75].

Displacement increased with RH due to water molecule-induced plasticisation in all cotton and groundwood samples (Figure 12B) [76]. During drying while returning to 20% RH, all samples experienced some shrinkage, demonstrated by displacement decrease. Not all the elongation that the sample experienced during the 80% RH condition was offset by this shrinkage, and the samples were permanently elongated by the end of RH cycling.

This section begins with discussion of the unaged, uncoated cotton and groundwood samples, followed by unaged LNP/MC-treated samples and MC only-treated samples. This is followed by discussion of the mechanical behaviour of these same samples when aged. Ultimately, LNP/MC composite coatings were more effective than MC alone for increasing stiffness in cotton samples, although less effective on groundwood. On cotton, after ageing, LNPs at any concentration also protected against the dimensional instability seen with the pure MC coating. All DMA-RH data pertaining to LNP/MC-treated samples can be found in Table 4 and Table 5.

#### 3.6.1. Unaged Controls

As shown in Table 4, unaged, uncoated cotton exhibited an initial storage modulus (*E′_20i_*) of 101 MPa at 20% RH, which decreased by 18.8% (Δ*E′_20i-80_*) to an *E′_80_* of 82 MPa at 80% RH due to moisture-induced plasticisation disrupting hydrogen bonding [76]. Groundwood was less affected, with the modulus decreasing by only 1.9% (Δ*E′_20i-80_*, from *E′_20i_* = 271 MPa to *E′_80_* = 266 MPa), indicating groundwood’s inherent hydrophobicity.

Displacement increased to 1.2% for cotton and 1.4% for groundwood (*d_20f_*; Table 4), suggesting elongation from RH cycling-induced mechanosorptive creep [28,77,78,79]. After returning to 20% RH, the modulus (*E′_20f_*) rose to 117 MPa (cotton) and 334 MPa (groundwood), indicating hornification (permanent stiffening), with Δ*E′_80-20f_* of 15.8% and 23.3%, respectively. This phenomenon may result from reduced fibrillation and fibre collapse [80], with implications for long-term flexibility and moisture resistance [28,81].

#### 3.6.2. Unaged MC Only-Coated Samples

Pure MC coatings increased stiffness in both substrates under dry conditions. Cotton’s *E′* rose from 101 MPa to 369 MPa after coating, a strong increase (265%). Groundwood’s *E′* rose from 271 MPa to 337 MPa, a moderate increase (24%) (Table 4). Hornification was up to 11.1% lower than MC alone for the MC/LNP composite treatment on groundwood (Table 4). However, displacement from the 2 wt% LNP treatment (*d_20f_* = 2.1) was more than double that of MC only-coated groundwood (*d_20f_* = 0.9) (Table 4). For cotton, displacement was higher with pure MC (*d_20f_* = 1.6) than when 2 wt% LNPs were incorporated (*d_20f_* = 1.3) (Table 4). This comparison of unaged pure MC coatings with the LNP/MC composite coatings shows that for groundwood paper, reduction in hornification came at the cost of higher displacement. This, plus a modest improvement in *E′* after adding LNPs to the MC (an increase of 94 to 108 MPa), means that LNP/MC composite-treated groundwood may not be a significant improvement over MC only-coated groundwood (Table 4).

#### 3.6.3. Unaged LNP/MC-Treated Samples

LNP/MC-treated cotton samples showed greater sensitivity to RH changes than uncoated controls (Table 4). For instance, Δ*E′_20i-80_* of 0.4% wt LNP/MC-treated cotton presents a modulus decrease by 25.1%, compared to 18.8% in uncoated cotton. LNP/MC-treated groundwood showed up to eightfold greater *E′* reduction than its uncoated counterpart, indicating increased hydrophilicity and susceptibility to plasticisation. For cotton, higher LNP concentrations improved stiffness across RH conditions. Notably, 2 wt% LNP/MC-treated cotton reached 584 MPa at 20% RH, over five times greater than uncoated cotton (101 MPa), demonstrating enhanced consolidation at the cost of increased hydrophilicity. Groundwood did not scale *E′* with increasing concentration (Table 4).

LNPs in MC improved hornification in groundwood and cotton papers and was reduced in the treated samples, as shown in Table 4. Cotton treated with 0.4 wt% and 2 wt% LNPs in MC showed no end-of-cycle stiffening, unlike uncoated cotton (Δ*E′_20f-80_* = 15.8%). Groundwood treated with LNPs in MC showed minimal hornification (Δ*E′_20f-80_* = 3.7% to 13.8%), unlike uncoated groundwood (Δ*E′_20f-80_* = 23.3%). Displacement data (Table 4) revealed that LNP/MC-treated groundwood, particularly at 2 wt% LNP/MC, had the highest post-cycling elongation (*d_20f_* = 2.1%), nearly twice that of the uncoated groundwood (*d_20f_* = 1.2%). This was possibly due to microcracking observed in SEM images (Figure 4B). These cracks may trap moisture, leading to irreversible deformation upon drying [82].

#### 3.6.4. Aged Controls

Table 5 summarises the RH-DMA findings of the aged controls and LNP/MC-treated samples. Aged and unaged cotton samples showed similar *E′* values and displacement patterns (Table S2). The exception was aged, uncoated groundwood, which stiffened by 11.6% at high RH, indicating resistance to moisture (Table S2). Therefore, any changes in aged, treated samples are likely to be due to the treatments rather than from ageing alone.

#### 3.6.5. Aged MC Only-Coated Samples

MC-coated samples were stiffer than the uncoated controls even after ageing, with *E′* doubling for cotton from 106 MPa to 297 MPa, and increasing by 41% for groundwood from 285 MPa to 401 MPa (Table 5). However, aged MC-coated cotton showed high displacement (2.6%) at the final 20% RH plateau. This displacement was much greater than both aged LNP/MC-treated cotton (1.1%, for both 0.4 wt% and 2 wt% LNP/MC) and aged uncoated cotton (1.3%) (Table 5). Aged MC-coated groundwood also showed a higher displacement (1.3%) relative to those of 1 wt% LNP/MC-treated groundwood (1.2%) and the uncoated control (1.1%) but not to the same extremes (Table 5). In aged cotton and groundwood samples, MC coatings continued to exhibit high stiffness, since MC possesses strong resistance to thermally-induced aging with little change to its degree of polymerisation [83], but introduced potential dimensional stability issues following RH cycling, especially in the cotton samples. The comparatively lower displacements for LNP/MC composite coatings for cotton suggests that LNPs offer some protection against dimensional instability in the long-term, even in environments with fluctuating RH. For conservation purposes, this is a key characteristic, as it may protect the treated paper against planar distortion and cockling.

#### 3.6.6. Aged LNP/MC-Treated Samples

In dry conditions, aged LNP/MC-treated cotton samples showed up to a 60.1% increase in *E′* compared to unaged samples, except for the 2 wt% LNP/MC treatment (Table 4 and Table 5). Groundwood either showed a reduced stiffness (up to 55.0%) or remained unchanged (Table 4 and Table 5). Hornification was reduced in aged cotton (up to 14.8% reduction in hornification for 1 wt% LNP/MC), but hornification increased in groundwood (up to 23.4% increase for 0.4 wt% LNP/MC) (Table 4 and Table 5). However, aged LNP/MC-treated groundwood showed good protection against increasing RH, with Δ*E′_20i-80_* as low as just 3.0%. Compared to aged uncoated controls, aged LNP/MC-treated cotton consistently showed lower displacement and higher stiffness. However, aged groundwood treated with 0.4 wt% and 2 wt% LNP/MC had lower *E′* than the aged control (242 and 200 MPa vs. 285 MPa), suggesting these concentrations may not be suitable for all long-term uses on groundwood (Table 5). In contrast, aged cotton treated with 0.4 wt% and 1 wt% LNP/MC showed promising mechanical properties.

## 4. Conclusions

This paper offers a preliminary study on the synthesis of sustainable LNPs and their application in methylcellulose (MC) as LNP/MC coatings on handmade model groundwood and cotton linter papers to assess their potential for conservation. FTIR analysis identified a lignin-associated peak at 1595 cm^−1^, which increased in cotton papers with LNP content. This trend was not observed in groundwood samples, which indicated uneven native lignin distribution that obscured LNP effects. On the other hand, the ratio of consistent peaks at 1635 cm^−1^ due to absorbed water bends and 1725 cm^−1^ due to carbonyl stretch identified in coated and uncoated groundwood samples suggested that an LNP/MC coating did not contribute to further carboxyl formation during ageing in the groundwood paper substrates. In this study, colour and pH changes were not found to correlate with LNP concentration, likely due to uneven lignin distribution and application variability. DMA-RH indicated that the LNP/MC coatings improved consolidation in cotton paper under dry conditions but were less effective on groundwood paper. LNP/MC coatings, however, may have had protective actions against the effect of increasing RH on aged groundwood paper, which mirrors how in the ATR/FTIR-derived oxidation index, 0.4 wt% LNP/MC treatment appeared to protect groundwood against carboxyl group formation during thermal ageing. LNP/MC-treated cotton may be more suitable over time to protect against dimensional instability than pure MC-coated cotton, since pure MC-coated cotton paper had high displacement, while the LNP/MC-treated cotton did not. Although current LNP/MC formulations require further optimisation for paper conservation, the promising findings of this preliminary study, such as colour stability, mechanical reinforcement, and the absence of LNP/MC-induced oxidative ageing of the paper substrates, warrant further investigation.

## Figures and Tables

**Table 1:** LNP size as determined from an SEM image of 0.4 wt% LNP/MC-coated groundwood paper (Figure 4B). Measurements were sorted into ten categories for interpretation. Most LNPs measured under 30.8 nm. Aggregated LNPs were disqualified from this sample set; only lone LNPs were considered.

Mean Diameter (nm)	Total LNPs Measured (%)
3.9–12.9	11.5
12.9–21.8	21.9
21.8–30.8	22.1
30.8–39.8	17.3
39.8–48.8	13.2
48.8–57.8	7.37
57.8–66.8	2.80
66.8–75.7	2.04
75.7–84.7	1.27
84.7–93.7	0.51

**Table 2:** ATR/FTIR spectra of unaged, uncoated cotton and groundwood, and unaged, 2 wt% LNP/MC-coated cotton and groundwood. Wavenumbers are a range to include slight peak shifts between the different samples. Abbreviations: *ν*, stretching vibration; *ν_as_*, asymmetric stretching vibration; *δ*, bending vibration; *δ_s_*, symmetric bending vibration; *ρ*, rocking vibration. See also Figure 8 for associated labelled spectra.

Wavenumber (cm^−1^)	Interpretation	Origin	Source
3332–3336	*ν*(O-H)	Cellulose, lignin	[60,61]
2899–2900, 2941–2944	*ν_as_*(C-H)	Lignin, MC, cellulose, hemicellulose	[62,63,64,65,66]
1722–1727	*ν*(C=O)	Lignin, hemicellulose	[66,67]
1636–1640	*ν*(C=O/C-O) (conjugated), bonding from absorbed water	Lignin, water	[63,66,68]
1592–1606	*ν*(C=C), *δ*(C-H)	Lignin	[64]
1509–1514	*ν*(C-C) and/or *ν*(C=C)	Lignin	[60,61,62,69,70]
1452–1461	*δ*(C-H)	Cellulose	[61]
1424–1428	*ν*(C-C), *δ*(C-H)	Lignin, cellulose	[61,62]
1361–1371	*δ_s_*(C-H), *ρ*(CH_2_)	Lignin, cellulose	[71]
1335	*δ*(C-H)	Cellulose	[61]
1315–1317	*ρ*(CH_2_)	Cellulose	[71]
1280	*δ*(C-H)	Cellulose (crystalline)	[72]
1265	*ν*(C-O)	Lignin	[61,62,64,69,70]
1205–1207	*ν*(C-O), *ν*(C-OH) or *δ*(C-CH)	Lignin, cellulose	[62,63,69,71]
1157–1161	*ν*(C-O), C-C ring breathing	Cellulose	[61,71]
1107–1109	*ν*(C-O), C-C ring breathing, *ν_as_*(C-O-C)	Lignin, cellulose	[60,70,71]
1000, 1030–1031, 1054–1055	*ν*(C-O), *ν*(C-O-C), *ν*(C-OH), in-plane *ρ*(–CH–)	Cellulose, hemicellulose	[60,61,70,71]
984–986, 895–897	*ν*(C-O-C)	Cellulose	[71]

**Table 3:** ATR/FTIR spectra of peaks not shared across the cohort of unaged, uncoated cotton and groundwood, and unaged, 2 wt% LNP/MC-coated cotton and groundwood. Frequencies are a range to include slight peak shifts between the different samples. Abbreviations: *ν*, stretching vibration; *ν_as_*, asymmetric stretching vibration; *δ*, bending vibration; *δ_s_*, symmetric bending vibration; *ρ*, rocking vibration. See also Figure 9.

Wavenumber (cm^−1^)	Interpretation	Extant in Which Samples	Origin	Source
1722–1727	*ν*(C=O)	Uncoated groundwood 2 wt% LNP/MC groundwood	Lignin, hemicellulose	[66,67]
1592–1606	*ν*(C=C), *δ*(C-H)	Uncoated groundwood 2 wt% LNP/MC cotton 2 wt% LNP/MC groundwood	Lignin	[64]
1509–1514	*ν*(C-C) and/or *ν*(C=C)	Uncoated groundwood 2 wt% LNP/MC cotton 2 wt% LNP/MC groundwood	Lignin	[60,61,62,69,70]
1280	*δ*(C-H)	Uncoated cotton 2 wt% LNP/MC cotton	(Crystalline) cellulose	[72]
1265	*ν*(C-O)	Uncoated groundwood 2 wt% LNP/MC groundwood	Lignin	[61,62,64,69,70]
1000	in-plane *ρ*(–CH–)	Uncoated cotton 2 wt% LNP/MC cotton	Cellulose	[71]

**Table 4:** *E′* and displacement (%) in initial 20% RH dry conditions, 80% RH humid conditions, and after returning to 20% RH conditions for the unaged LNP/MC-treated samples. The unaged, uncoated controls are provided for comparison.

Sample	*E′_20i_* * (MPa)	*E′_80_* (MPa)	*E′_20f_* (MPa)	Δ*E′_20i-80_* (%)	Δ*E′_20i-20f_* (%)	*d_20i_* (%)	*d_20f_* (%)
Cotton, uncoated, unaged	101	82	117	−18.8	+15.8	0.1	1.4
Groundwood, uncoated, unaged	271	266	334	−1.9	+23.3	0.2	1.2
Cotton, MC only, unaged	369	292	383	−20.9	+3.8	0.1	1.6
Groundwood, MC only, unaged	337	332	421	−1.5	+24.9	0.1	0.9
Cotton, 0.4 wt% LNP/MC, unaged	346	259	312	−25.1	−9.8	0.1	1.3
Cotton, 1 wt% LNP/MC, unaged	401	324	455	−19.2	+13.5	0.1	1.9
Cotton, 2 wt% LNP/MC, unaged	584	450	566	−24.6	−8.7	0.1	1.3
Groundwood, 0.4 wt% LNP/MC, unaged	445	375	479	−15.7	+7.6	0.1	1.8
Groundwood, 1 wt% LNP/MC, unaged	431	368	447	−14.6	+3.7	0.1	1.4
Groundwood, 2 wt% LNP/MC, unaged	444	391	522	−11.4	+13.8	0.2	2.1

* (*E′_20i_* at initial 20% RH, *E′_80_* at 80% RH, *E′_20f_* at final 20% RH, *d_20i_* percent displacement at initial 20% RH, *d_20f_* percent displacement at final 20% RH). Standard deviation (SD) for *E′_20i_* cotton samples = ±55.2; groundwood = ±29.7. SD for *E′_80_* cotton samples = ±28.3; groundwood = ±29.7. SD for *E′_20f_* cotton samples = ±4.24; groundwood = ±10.6. SD for *d_20i_* cotton samples = ±2.83 × 10^−2^; groundwood = ±7.07 × 10^−2^. SD for *d_20f_* cotton samples = ±7.07 × 10^−3^; groundwood = ±5.66 × 10^−2^. This applies to Table 4, Table 5 and Table S2.

**Table 5:** *E′* and displacement (%) in initial 20% RH dry conditions, 80% RH humid conditions, and after returning to 20% RH conditions for aged LNP/MC-treated samples. Aged controls are provided for comparison.

Sample	*E′_20i_* (MPa)	*E′_80_* (MPa)	*E′_20f_* (MPa)	Δ*E′_20i-80_* (%)	Δ*E′_20i-20f_* (%)	*d_20i_* (%)	*d_20f_* (%)
Cotton, uncoated, aged	106	88	122	−17.0	+15.1	0.1	1.3
Groundwood, uncoated, aged	285	318	368	+11.6	+29.1	0.1	1.1
Cotton, MC only, aged	297	244	317	−17.9	+6.7	0.1	2.6
Groundwood, MC only, aged	401	398	436	−0.8	+8.7	0.2	1.3
Cotton, 0.4 wt% LNP, aged	453	350	452	−22.7	−0.2	0.0	1.1
Cotton, 1 wt% LNP/MC, aged	642	487	634	−24.1	−1.3	0.1	0.8
Cotton, 2 wt% LNP/MC, aged	470	363	456	−22.8	−3.0	0.1	1.1
Groundwood, 0.4 wt% LNP/MC, aged	242	230	317	−5.0	+31.0	0.1	0.9
Groundwood, 1 wt% LNP/MC, aged	437	424	502	−3.0	+14.9	0.1	1.2
Groundwood, 2 wt% LNP/MC, aged	200	193	266	−3.5	+33.0	0.2	1.0

## Data Availability

The original contributions presented in this study are included in the article and Supplementary Material. Further inquiries can be directed to the corresponding authors.

## Supplementary Materials

The following supporting information can be downloaded at: https://www.mdpi.com/article/10.3390/polym17212934/s1, Table S1: ΔE_00_ compared to unaged reference sample for each treatment; Table S2: *E′* and displacement (%) in initial 20% RH dry conditions, 80% RH humid conditions, and after returning to 20% RH conditions for aged and unaged uncoated control samples.
