# Supplementary material for: A Multi-Analytical Study of Nanolignin/Methylcellulose-Coated Groundwood and Cotton Linter Model Papers
Source: Polymers (Basel). 2025 Oct 31;17(21):2934. doi: 10.3390/polym17212934 (PMC12609468; doi:10.3390/polym17212934)
Supplement: Supplementary file 1 [file polymers-17-02934-s001.zip › polymers-3942688-supplementary.pdf]

A multi-analytical study of nanolignin/methylcellulose-coated groundwood and cotton linter model papers

Mia Bloss,[1] Marianne Odlyha,[2] Charis Theodorakopoulos,[1,*]

[1] Science in Conservation of Fine Art, School of Design, Arts and Creative Industries, Northumbria University, Newcastle upon Tyne NE1 8ST, UK.
[2] School of Natural Sciences, Birkbeck, University of London, London WC1E 7HX, UK.

SUPPLEMENTARY MATERIALS

Table S1. ΔE_{00} compared to the unaged reference sample for each treatment, where $k_C = k_H = 1$ and k_L varies. Values in red exceed dE00 = 2.0, indicating noticeable colour difference.

Sample		$k_L = 1$	$k_L = 1.5$	$k_L = 2$
Cotton	Uncoated, 24h	0.90	0.88	0.87
	Uncoated, 48h	1.41	1.40	1.40
	Uncoated, 72h	1.77	1.77	1.76
	Uncoated, 144h	2.58	2.57	2.57
	Uncoated, 312h	2.12	2.11	2.11
	Uncoated, 384h	2.74	2.73	2.72
	MC only, 24h	0.71	0.68	0.67
	MC only, 48h	0.88	0.85	0.83
	MC only, 72h	1.46	1.40	1.38
	MC only, 144h	1.94	1.93	1.93
	MC only, 312h	3.88	3.75	3.71
	MC only, 384h	2.24	2.10	2.05
	0.4 wt% LNP, 24h	2.25	1.81	1.63
	0.4 wt% LNP, 48h	2.31	1.89	1.72
	0.4 wt% LNP, 72h	2.25	1.76	1.55
	0.4 wt% LNP, 144h	2.35	1.78	1.53
	0.4 wt% LNP, 312h	0.67	0.65	0.65
	0.4 wt% LNP, 384h	0.61	0.52	0.48
	1 wt% LNP, 24h	1.77	1.49	1.38
	1 wt% LNP, 48h	1.54	1.54	1.54
	1 wt% LNP, 72h	2.31	1.93	1.78
	1 wt% LNP, 144h	2.00	1.53	1.33
	1 wt% LNP, 312h	3.52	2.35	1.77
	1 wt% LNP, 384h	2.64	1.78	1.35
	2 wt% LNP, 24h	6.70	4.72	3.79
	2 wt% LNP, 48h	1.58	1.42	1.36

	2 wt% LNP, 72h	2.17	1.73	1.54
	2 wt% LNP, 144h	1.12	1.08	1.06
	2 wt% LNP, 312h	0.71	0.70	0.70
	2 wt% LNP, 384h	0.92	0.82	0.78
**Ground-wood**	Uncoated, 24h	1.62	1.60	1.59
	Uncoated, 48h	2.42	2.38	2.36
	Uncoated, 72h	3.00	2.99	2.99
	Uncoated, 144h	2.81	2.73	2.70
	Uncoated, 312h	3.04	3.00	2.98
	Uncoated, 384h	4.08	3.96	3.92
	MC only, 24h	1.92	1.88	1.86
	MC only, 48h	2.28	2.24	2.23
	MC only, 72h	2.85	2.77	2.74
	MC only, 144h	3.41	3.34	3.32
	MC only, 312h	3.49	3.34	3.29
	MC only, 384h	3.65	3.55	3.52
	0.4 wt% LNP, 24h	1.73	1.64	1.61
	0.4 wt% LNP, 48h	1.49	1.49	1.49
	0.4 wt% LNP, 72h	1.47	1.47	1.47
	0.4 wt% LNP, 144h	2.75	2.65	2.62
	0.4 wt% LNP, 312h	2.19	2.17	2.16
	0.4 wt% LNP, 384h	2.77	2.67	2.63
	1 wt% LNP, 24h	2.45	2.11	1.98
	1 wt% LNP, 48h	4.11	3.26	2.91
	1 wt% LNP, 72h	2.20	2.04	1.99
	1wt% LNP, 144h	2.38	2.19	2.12
	1 wt% LNP, 312h	1.79	1.78	1.78
	1 wt% LNP, 384h	2.23	2.21	2.21
	2 wt% LNP, 24h	1.95	1.77	1.71
	2 wt% LNP, 48h	2.83	2.31	2.10
	2 wt% LNP, 72h	3.68	2.81	2.43
	2 wt% LNP, 144h	2.62	2.17	1.99
	2 wt% LNP, 312h	0.91	0.91	0.90
	2 wt% LNP, 384h	0.80	0.79	0.79

Table S2. *E'* and displacement (%) in initial 20% RH dry conditions, 80% RH humid conditions, and after returning to 20% RH conditions for the aged and unaged uncoated control samples.

Sample	E'_{20i} (MPa)	E'_{80} (MPa)	E'_{20f} (MPa)	$\Delta E'_{20i\text{-}80}$ (%)	$\Delta E'_{20i\text{-}20f}$ (%)	d_{20i} (%)	$\%d_{20f}$ (%)
Cotton, uncoated, aged	106	88	122	-17.0	+15.1	0.1	1.3
Groundwood, uncoated, aged	285	318	368	+11.6	+29.1	0.1	1.1
Cotton, uncoated, unaged	101	82	117	-18.8	+15.8	0.1	1.4
Groundwood, uncoated, unaged	271	266	334	-1.9	+23.3	0.2	1.2
